# Effect of Repeated Whole Blood Donations on Aerobic Capacity and Hemoglobin Mass in Moderately Trained Male Subjects: A Randomized Controlled Trial

**DOI:** 10.1186/s40798-016-0067-7

**Published:** 2016-11-22

**Authors:** Julie Meurrens, Thomas Steiner, Jonathan Ponette, Hans Antonius Janssen, Monique Ramaekers, Jon Peter Wehrlin, Philippe Vandekerckhove, Louise Deldicque

**Affiliations:** 1 Exercise Physiology Research Group, Department of Kinesiology, KU Leuven, Leuven, Belgium; 2 Section for Elite Sport, Swiss Federal Institute of Sports, Magglingen, Switzerland; 3 Belgian Red Cross-Flanders, Mechelen, Belgium; 4 Faculty of Medicine, University of Ghent, Ghent, Belgium; 5 Department of Public Health and Primary Care, Faculty of Medicine, KU Leuven, Leuven, Belgium; 6 Institute of Neuroscience, Université catholique de Louvain, 1, Place Pierre de Coubertin box L08.10.01, 1348 Louvain-la-Neuve, Belgium

**Keywords:** Iron, Erythropoietin, Ferritin, Maximal oxygen consumption, Lactate, Maximal power output

## Abstract

**Background:**

The aims of the present study were to investigate the impact of three whole blood donations on endurance capacity and hematological parameters and to determine the duration to fully recover initial endurance capacity and hematological parameters after each donation.

**Methods:**

Twenty-four moderately trained subjects were randomly divided in a donation (*n* = 16) and a placebo (*n* = 8) group. Each of the three donations was interspersed by 3 months, and the recovery of endurance capacity and hematological parameters was monitored up to 1 month after donation.

**Results:**

Maximal power output, peak oxygen consumption, and hemoglobin mass decreased (*p* < 0.001) up to 4 weeks after a single blood donation with a maximal decrease of 4, 10, and 7%, respectively. Hematocrit, hemoglobin concentration, ferritin, and red blood cell count (RBC), all key hematological parameters for oxygen transport, were lowered by a single donation (*p* < 0.001) and cumulatively further affected by the repetition of the donations (*p* < 0.001). The maximal decrease after a blood donation was 11% for hematocrit, 10% for hemoglobin concentration, 50% for ferritin, and 12% for RBC (*p* < 0.001). Maximal power output cumulatively increased in the placebo group as the maximal exercise tests were repeated (*p* < 0.001), which indicates positive training adaptations. This increase in maximal power output over the whole duration of the study was not observed in the donation group.

**Conclusions:**

Maximal, but not submaximal, endurance capacity was altered after blood donation in moderately trained people and the expected increase in capacity after multiple maximal exercise tests was not present when repeating whole blood donations.

**Electronic supplementary material:**

The online version of this article (doi:10.1186/s40798-016-0067-7) contains supplementary material, which is available to authorized users.

## Key Points

- Maximal power output, VO_2_peak, and hemoglobin mass were decreased up to 4 weeks after a single blood donation in moderately trained people
- Beneficial training adaptations seemed somewhat lowered by repeated whole blood donations
- Key hematological parameters for oxygen transport were lowered by a single donation and cumulatively further affected by their repetition
- Measures to counteract the alterations in hematological parameters should be developed to minimize the impact on endurance capacity and thereby to attract more athletes to become donors
- Athletes constitute a very healthy potential donor population and should consider becoming plasma donors as there also is an increased need for plasma worldwide and plasma donation does not affect their hemoglobin levels at all

## Background

The demand for blood and blood products evolves due to different factors. On the one hand, blood transfusion triggers have become stricter reducing the need for the transfusion of red cells [1]. On the other hand, as a result of medical progress, more red cells are required [2, 3] due to more complex surgical procedures, increasing number of transplantations, advances in hematology and oncology for which transfusions are necessary [3–5], and aging of the population [6, 7] with increasing rates of medical interventions with old age [8]. Demand for plasma and plasma-derived products such as coagulation factors, albumin, and immunoglobulins increases worldwide mainly due to new indications and an increased utilization in countries as they become more developed.

On the donor side, more and more tightened selection criteria exist [9–11], making finding enough donors a continuous effort. The World Health Organization drawn up many strategies and campaigns to encourage people to become or stay blood donors. Despite the efforts, these initiatives do not always attract the preferred population [12], which consists of voluntary, unpaid blood donors who give blood and plasma mainly for altruistic reasons [2], and seems to be the healthiest group because of their lower risk of blood-borne diseases [2]. From this perspective, sport athletes would be an interesting donor population to be recruited. However, due to the potential detrimental effects on sport performance, athletes are not easily convinced to give blood on a regular basis. It is therefore essential to better understand how blood donation impacts sport performance and, in turn, to inform athletes adequately.

After donating one unit of whole blood, hematological parameters decrease immediately [13–15]. Blood withdrawal causes a reduction of plasma volume which is almost completely restored 24–48 h after donation [14]. Recovery of hematocrit, ferritin, and hemoglobin concentrations takes more time as those parameters have been found to be reduced beyond 4 weeks after donation [15], which corresponds approximately to the period needed for total hemoglobin mass to return to baseline levels [16]. Of note, serum ferritin concentration decreases further when donations are repeated [17]. While the effect of one blood donation on hematological parameters is quite well documented, there is a paucity of data after repeated blood donations while donors are invited to give blood three or four times a year. As already evidenced for serum ferritin, it can be hypothesized that changes in hematological parameters are more severe when donations are repeated regularly, which could lead to iron deficiency.

As the aforementioned hematological parameters have a critical role in the regulation of oxygen-carrying capacity and thereby maximal oxygen uptake (VO_2_max), blood donation is expected to negatively impact endurance performance [18–20]. In 1995, Panebianco et al. showed that blood donation reduced maximal performance in cyclists for at least 1 week, while submaximal performance was not affected [21]. Other studies confirmed that VO_2_max was reduced after blood donation from a few hours and days [20–23] until 2 weeks [24]. Blood donation does not only reduce VO_2_max as time to exhaustion has been shown to be altered as well [13]. Taken together, those results show that VO_2_max and, more broadly, maximal endurance performance is reduced after a blood donation. However, the conclusions of the few studies looking at recovery of performance or VO_2_max after donation have to be taken with caution as no placebo group was included, which preclude from distinguishing the effects of donation itself and the effect of repeating strenuous exercise for determining maximal parameters.

Although several studies analyzed the influence of whole blood donation on endurance performance, there is a paucity of data reporting the time necessary to fully recover. Most of the studies stopped data collection after a few days. Furthermore, whereas most donors usually give blood three or four times a year, no study has evaluated the effect of repeated donations on endurance performance. Therefore, the aims of the present study were: (1) to investigate the impact of three donations on endurance capacity and hematological parameters and (2) to determine the duration to fully recover initial endurance capacity and hematological parameters after each donation. A placebo group was included to isolate the effects of blood donation from those of repeated strenuous exercise.

## Methods

### Subjects

**Fig. 1:**
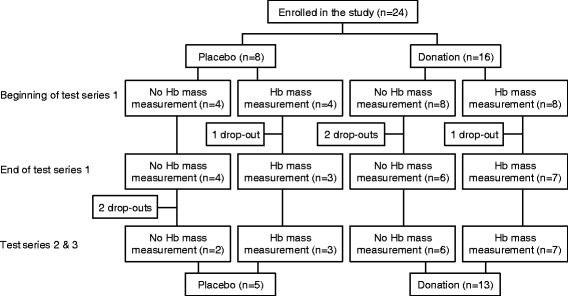
Subject flow chart

A statistical power analysis was performed to determine the optimal number of subjects needed to find a difference in the mean of 15% (expected for VO_2_max) with a standard deviation corresponding to 20% of the means with a power of 80%. Twenty-four young men (*n* = 8 in the placebo group and *n* = 16 in the donation group, randomly assigned) volunteered to participate in a longitudinal study (Table 1) (Fig. 1). Inclusion criteria were as follows: recreational sportsmen (1 to 6 h sport a week), age 18 to 30 years, BMI 20 to 28 kg⋅m^2 -1^, and no contraindication to perform exercise at maximal intensity. Subjects were instructed to refrain from vigorous physical activity 2 days before every experimental session and from alcohol and smoking 1 day before. During the whole duration of the study, subjects were asked to maintain their habitual lifestyle, i.e., physical activity and diet. Furthermore, all participants came to the laboratory around the same time to minimize diurnal variations. All participants provided written informed consent after explaining all potential risks of the study and the right to withdraw at any time. The study was approved by the Ethics Committee of the KU Leuven and the investigation was performed according to the principles outlined in the Declaration of Helsinki.

**Table 1:** Subject characteristics at the start of the study

	Placebo	Donation	*p* value
Age (years)	27.2 ± 4.35	25.6 ± 0.76	0.62
Weight (kg)	75.1 ± 4.51	73.8 ± 1.96	0.75
Height (cm)	179.5 ± 1.67	180.2 ± 1.82	0.81
BMI (kg/m^2^)	23.2 ± 1.06	22.7 ± 0.49	0.64
Sport/week (h)	5.1 ± 0.74	3.9 ± 0.55	0.22
VO_2_peak (ml/kg^/^min)	56.9 ± 4.63	56.7 ± 1.45	0.97

Values are means ± SEM. *n* = 8 in placebo and *n* = 16 in donation

Four subjects (one in the placebo group and three in the donation group) gave up before the end of the first set of experiments. As they were incomplete, their data were not included in the analyses, resulting in a total number of subjects of 20 for the first set of experiments. Two additional subjects in the placebo group gave up before the end of the second set of experiments, resulting in a total of 18 subjects for the second and the third sets of experiments. Five subjects gave up due to the high time-consuming character of the study and one subject got injured independently of the study.

### Exercise Testing

**Fig. 2:**
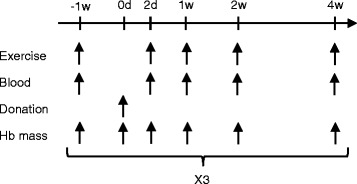
Schematic study design

One week before the first test series (-1w), subjects reported to the laboratory to perform a maximal incremental test (Fig. 2). Weight and height were measured. After 10 min in sitting position, three blood samples were taken in an antecubital vein (one in a 10-ml clot activator tube, one in a 4.5-ml lithium heparin tube, and one in a 3-ml EDTA tube). Fifteen minutes after blood sampling, a peak oxygen consumption (VO_2_peak) test was performed on a bicycle ergometer (Avontronic, Cyclus 2, Leipzig, Germany) in a ventilated laboratory with a constant temperature of 18 °C. Seat height was recorded during the first test and remained constant throughout the 18 tests. Subjects were also blinded for time, power, and heart rate. The protocol started at 70 W, followed by incremental loads of 30 W every 2 min until exhaustion. The maximal power output (Pmax) was calculated as the last step completed plus the last increment corrected for the sustained duration, which corresponded to the Total Time of the test. Cadence must be maintained at 85–90 rpm. Oxygen consumption (VO_2_), carbonic dioxide production (VCO_2_) (Metalyzer II, Cortex, Leipzig, Germany) as well as heart rate (HR, Polar, Kempele, Finland) were continuously monitored during the test. Oxygen pulse was calculated by dividing VO_2_peak and HRmax at the end of the test. Blood lactate was measured before, during (at 190 W), and at the end of the test by taking a capillary blood sample (5 μl) from an earlobe (Lactate Pro, Arkray, Japan). Subjects were not verbally encouraged during the entire test. The VO_2_peak test and blood sampling were repeated 2 days (2d), 1 week (1w), 2 weeks (2w), and 4 weeks (4w) after each of the three blood donations (see below) following exactly the same conditions (Fig. 2). Due to ethical considerations, the VO_2_peak test was not performed the day of the donation (0d).

### Hemoglobin Mass Measurement

Due to practical issues, hemoglobin mass was determined on half of the subjects (*n* = 3 in placebo and *n* = 7 in donation). Hemoglobin mass was measured 45 min after the end of the incremental test to ensure that ventilation had returned to basal levels using a modified version [25] of the optimized carbon monoxide (CO) rebreathing method developed by Schmidt and Prommer [26]. This method consists in taking capillary earlobe blood samples (70 μl) for the determination of carboxyhemoglobin concentrations (ABL 90 Flex Radiometer, Zoetermeer, The Netherlands). Five samples were taken at basal and averaged and two other samples were taken 6 and 8 min after the initial inhalation of the CO dose. A CO dose of 1.0 ml⋅kg^−1^ body mass was administered and rebreathed for 2 min trough a glass spirometer (Blood tec, Bayreuth, Germany). Before and 4 min after the initial inhalation of CO, end-tidal CO concentration in the lungs was measured (PAC 5500 CO, Dräger, Wemmel, Belgium). At the end of the test, the remaining amount of CO located in the system (spirometer + breathing bag) was determined. The hemoglobin mass was calculated and reported relative to body weight according to Steiner and Wehrlin [25, 27]. The measurements were repeated at -1w, 0d, 2d, 1w, 2w, and 4w from each blood donation.

### Blood Donation

One week after exercise pre-testing (0d), participants reported to the Red Cross Center in Leuven (Belgium). They underwent a blood donation of 470 ml according to the Belgian Law of 01/02/2005 (Donation) or had a similar sensation of undergoing a blood donation (placebo) as described in Howell et al [28]. During each donation or simulation of donation, the subjects were blinded and they were listening to music through a headset. To study the repetitive effect, blood donation was repeated after 3 months (second test series) and after 6 months (third test series).

### Blood Analyses

The lithium heparin and the EDTA tubes were analyzed within 24 h following blood drawing in the laboratory of the university hospital (Leuven). The following parameters were analyzed using an automated device (Sysmex XE-5000^TM^, Hoeilaart, Belgium): blood platelets, hematocrit, hemoglobin concentration, mean corpuscular hemoglobin (MCH), mean corpuscular hemoglobin concentration (MCHC), mean corpuscular volume (MCV), mean platelet volume (MPV), red blood cell count (RBC), red blood cell distribution width (RDW), white blood cell count (WBC), ferritin, iron, transferrin, and transferrin saturation. The clot activator tubes were centrifuged at the end of each experimental day for 6 min at 3000 g at 4 °C. The supernatant was collected and stored at −80 °C. Serum erythropoietin was determined using the Human Erythropoietin Elisa Kit (Abcam, Cambridge, UK).

### Statistical Analysis

All values are expressed as mean ± SEM. A mixed ANOVA model (SAS Statistical Software) was used with the subjects as a random variable and groups (placebo and donation) and condition (time) as fixed independent variables. As a group effect was only observed for hematocrit, hemoglobin, ferritin, and RBC, a mixed ANOVA model with the time as a fixed independent variable was applied to the placebo and to the donation group separately (Table 2). When appropriate, contrast analyses were performed to compare means. Statistical significance was set at *p* < 0.05.

**Table 2:** *F* and *p* values from the global ANOVA analyses

	All subjects				Placebo		Donation	
	Group		Time		Time		Time	
	*F* value	*p* value	*F* value	*p* value	*F* value	*p* value	*F* value	*p* value
Pmax	0.43	0.5219	9.54	<0.0001	4.13	<0.0001	6.48	<0.0001
VO_2_peak	0.32	0.5775	4.31	<0.0001	0.81	0.6573	5.46	<0.0001
Hb mass	0.80	0.3959	3.82	<0.0001	0.51	0.9255	6.03	<0.0001
EPO	0.10	0.7631	2.88	0.0003	0.76	0.7181	2.62	0.0019
Lactate 190W	0.18	0.6722	9.10	<0.0001	8.34	0.0008	3.22	<0.0001
Lactate post	0.01	0.9069	4.12	<0.0001	1.62	0.0993	3.63	<0.0001
HR max	0.21	0.6510	4.44	<0.0001	1.75	0.0699	3.70	<0.0001
Oxygen pulse	0.41	0.5325	1.61	0.0744	0.61	0.8459	3.57	<0.0001
VE max	0.09	0.7675	3.78	<0.0001	1.23	0.2807	3.15	0.0002
Total time	0.43	0.5241	9.61	<0.0001	4.12	<0.0001	6.57	<0.0001
Hematocrit	12.90	0.0021	6.51	<0.0001	1.56	0.1168	12.38	<0.0001
Hemoglobin	6.77	0.0180	8.48	<0.0001	1.25	0.2635	12.94	<0.0001
Iron	0.55	0.4670	3.30	0.0003	1.69	0.0811	2.91	0.0006
Ferritin	8.49	0.0093	3.48	<0.0001	1.12	0.3589	6.50	<0.0001
Transferrin	1.98	0.1769	2.97	0.0003	0.96	0.5055	3.11	0.0003
Transf sat	1.25	0.2785	2.98	0.0003	1.62	0.0976	3.10	0.0003
MCH	0.03	0.8603	6.36	<0.0001	1.82	0.0551	6.61	<0.0001
MCHC	0.01	0.9105	8.10	<0.0001	2.64	0.0045	6.81	<0.0001
MCV	0.03	0.8689	4.14	<0.0001	1.35	0.2047	4.26	<0.0001
MPV	2.77	0.1136	2.47	0.0028	1.11	0.3699	2.71	0.0013
RBC	6.93	0.0169	6.31	<0.0001	1.18	0.3122	11.67	<0.0001
RDW	1.05	0.3181	10.74	<0.0001	2.02	0.0303	10.70	<0.0001
WBC	0.89	0.3589	0.86	0.5990	0.46	0.9445	1.61	0.0821
Platelets	0.09	0.7639	3.37	<0.0001	1.43	0.1670	3.14	0.0002

*Pmax* maximal power output, *VO*_*2*_ *peak* maximal aerobic consumption, *Hb mass* hemoglobin mass, *EPO* erythropoietin, *HR* heart rate, *VE* ventilation, *Transf sat* transferrin saturation, *MCH* mean corpuscular hemoglobin, *MCHC* mean corpuscular hemoglobin concentration, *MCV* mean corpuscular volume, *MPV* mean platelet volume, *RBC* red blood cell count, *RDW* red blood cell distribution width, *WBC* white blood cell count

## Results

### Single Versus Repeated Whole Blood Donations Differently Alter Endurance Capacity

**Fig. 3:**
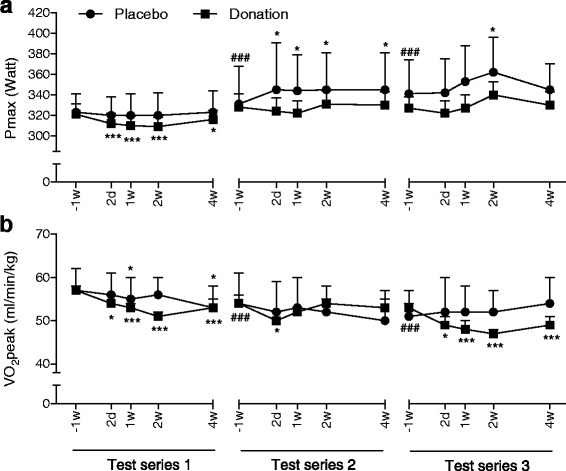
Maximal power output and peak oxygen consumption. Evolution of the maximal power output (*Pmax*, **a**) and peak oxygen consumption (*VO*_*2*_ *peak*, **b**) before and after each of the three test series in the placebo and donation group. Data are expressed as means ± SEM. *n* = 7 in placebo and *n* = 13 in donation for test series 1; *n* = 5 in placebo and *n* = 13 in donation for test series 2 and 3. ^###^ *p* < 0.001 vs. -1w of test series 1, same group. *p < 0.05, ***p < 0.001 vs. -1w of the same test series, same group. *Symbols* are placed above the curves for the placebo group and under the curves for the donation group

Pmax decreased by 3% 2d, by 4% 1w, and 2w and by 2% 4w after the first blood donation while remaining constant in the placebo group (Fig. 3a). In the placebo group, a training effect was observed starting from the second test series as pre-values from the second (+3%) and the third test series (+5%) were higher compared to the pre-values from the first one. Within the placebo group, Pmax was higher at 2d, 1w, 2w, and 4w than at -1w in test series 2 and at 2w in test series 3. This training effect was not observed in the donation group as Pmax did not increase with time. Logically, the results for total time paralleled the results of Pmax (Table 3). VO_2_peak decreased by 5% 2d, by 7% 1w, by 10% 2w, and by 7% 4w after the first blood donation (Fig. 3b). VO_2_peak also decreased in the placebo group after 1w (−5%) and 4w (−8%) in test series 1, with no further modification in test series 2 and 3. In the donation group, pre-values of VO_2_peak were lower in test series 2 and 3 compared to pre-values in test series 1. Within test series 2, VO_2_peak decreased only 2d after donation (−4%) and returned to basal values 1w after. In test series 3, VO_2_peak decreased by 7% 2d, by 9% 1w, by 11% 2w, and by 7% 4w after blood donation. In summary, the first blood donation had a higher negative effect on endurance capacity than the second and the third donations as both Pmax and VO_2_peak decreased after the first blood donation while only VO_2_peak decreased after the second and third donations. Our data also indicate that the positive training effect on Pmax observed in the placebo group during the second and the third test series was not present in the donation group.

**Table 3:** Effect of repeated blood donations on exercise parameters

		Test series 1					Test series 2					Test series 3				
		-1w	2d	1w	2w	4w	-1w	2d	1w	2w	4w	-1w	2d	1w	2w	4w
Lactate 190W	P	3.8 ± 1.1	3.2 ± 1.0	3.5 ± 1.1	3.5 ± 1.2	3.5 ± 1.2	2.1 ± 0.8#	3.1 ± 1.5	3.0 ± 1.4*	2.9 ± 1.2*	3.1 ± 1.4*	2.8 ± 1.1##	2.6 ± 1.0	2.2 ± 0.9	2.1 ± 0.7*	1.7 ± 0.3*
(mmol/l)	D	2.8 ± 0.3	3.1 ± 0.3	3.0 ± 0.4	3.0 ± 0.3	2.6 ± 0.4	2.0 ± 0.2##	2.3 ± 0.3*	2.5 ± 0.3**	2.1 ± 0.3	2.1 ± 0.2	2.4 ± 0.4###	2.1 ± 0.3	2.0 ± 0.3*	1.8 ± 0.3**	1.7 ± 0.2***
Lactate post	P	11.9 ± 0.8	12.1 ± 0.7	11.6 ± 0.2	13.6 ± 1.1	13.2 ± 1.9	9.3 ± 0.9	10.2 ± 2.3	10.7 ± 1.8	9.7 ± 0.9	11.2 ± 1.2	10.2 ± 1.3	9.8 ± 0.7	10.3 ± 1.3	9.8 ± 1.1	8.7 ± 1.0
(mmol/l)	D	13.6 ± 0.8	13.0 ± 1.0	12.1 ± 1.1	11.8 ± 1.0	10.8 ± 0.6**	11.5 ± 1.1##	10.9 ± 0.9	11.0 ± 1.1	10.8 ± 0.9	10.6 ± 0.8	11.8 ± 1.0###	10.2 ± 1.3	9.5 ± 1.2*	10.1 ± 0.7	9.2 ± 0.9*
HR max	P	184 ± 4	183 ± 3	179 ± 5*	178 ± 6*	177 ± 4**	169 ± 7#	174 ± 5*	174 ± 4*	178 ± 4**	175 ± 5*	176 ± 4	174 ± 5	177 ± 4	180 ± 5	177 ± 4
(bpm)	D	187 ± 4	187 ± 3	183 ± 3*	180 ± 3***	182 ± 3**	180 ± 4#	181 ± 3	182 ± 2	182 ± 2	180 ± 3	181 ± 3###	178 ± 3	175 ± 5	181 ± 3	179 ± 4
Oxygen pulse	P	0.31 ± 0.02	0.31 ± 0.03	0.30 ± 0.03	0.31 ± 0.03	0.29 ± 0.03	0.32 ± 0.04	0.30 ± 0.03	0.31 ± 0.04	0.29 ± 0.03	0.28 ± 0.03	0.29 ± 0.03	0.30 ± 0.04	0.29 ± 0.03	0.29 ± 0.03	0.30 ± 0.04
(ml O_2_/beat/kg)	D	0.31 ± 0.01	0.30 ± 0.02	0.29 ± 0.01*	0.29 ± 0.01**	0.29 ± 0.01*	0.30 ± 0.01##	0.28 ± 0.01*	0.29 ± 0.01	0.30 ± 0.01	0.30 ± 0.01	0.29 ± 0.01##	0.27 ± 0.01*	0.28 ± 0.01*	0.26 ± 0.01**	0.27 ± 0.01*
VE max	P	139 ± 11	132 ± 14	130 ± 15	125 ± 15	124 ± 11*	131 ± 8	119 ± 10	133 ± 10	136 ± 12	137 ± 17	138 ± 16	134 ± 11	139 ± 12	147 ± 11*	139 ± 13
(l/min)	D	135 ± 7	135 ± 6	130 ± 6	128 ± 9	126 ± 8	123 ± 7#	122 ± 7	131 ± 7*	137 ± 7**	126 ± 6	145 ± 7	131 ± 8*	134 ± 7*	143 ± 7	135 ± 6
Total time	P	16.9 ± 1.2	16.7 ± 1.2	16.7 ± 1.4	16.7 ± 1.5	16.9 ± 1.4	17.4 ± 2.4#	18.3 ± 3.1*	18.2 ± 2.3*	18.3 ± 2.4*	18.4 ± 2.4*	18.0 ± 2.2###	18.1 ± 2.2	18.9 ± 2.4	19.5 ± 2.2*	18.3 ± 1.7
(min)	D	16.9 ± 0.9	16.1 ± 1.0**	16.2 ± 0.8***	16.3 ± 0.8***	16.5 ± 0.9*	17.7 ± 1.1	17.2 ± 1.2*	16.9 ± 1.2	17.9 ± 1.2	17.8 ± 1.2	17.4 ± 1.1	16.8 ± 1.1	17.7 ± 1.1	18.5 ± 1.2	17.1 ± 0.9

Values are means ± SEM (*n* = 7 in *P* and *n* = 13 in *D* for test series 1; *n* = 5 in P and *n* = 13 in *D* for test series 2 and 3. *HR* heart rate, *VE* ventilation, *P* placebo, *D* donation. ^#^ *p* < 0.05, ^##^ *p* < 0.01, ^###^ *p* < 0.001 vs. -1w of test series 1, same group. *p < 0.05, **p < 0.01, ***p < 0.001 vs. -1w of the same test series, same group

### Whole Blood Donation Does Not Modify Lactate, Heart Rate, or Ventilation

Submaximal lactate values at 190 W decreased steadily (Table 2) from the first (3.8 mM in placebo and 2.8 mM in donation, Table 3) to the 15th exercise test (1.7 mM in each group) with no difference between the groups, showing a similar training effect at submaximal intensity in both groups. A general decrease across time was also observed for post-exercise lactate values but the decrease was more pronounced in the donation group (from 13.6 to 9.2 mM, *p* < 0.001) compared to the placebo group (from 11.9 to 8.7 mM, *p* = 0.0993) (Tables 2 and 3). Maximal heart rate recorded at the end of the exercise test decreased with time in the donation group and tended to decrease in the placebo group as well (*p* = 0.0699, Table 2). Oxygen pulse decreased over time and within each donation in the donation group only (Tables 2 and 3). The maximal ventilation showed some fluctuation over time but no clear training effect or any effect of donation could be observed (Table 3).

### Whole Blood Donation Reduces Hemoglobin Mass and Increases Serum Erythropoietin

**Fig. 4:**
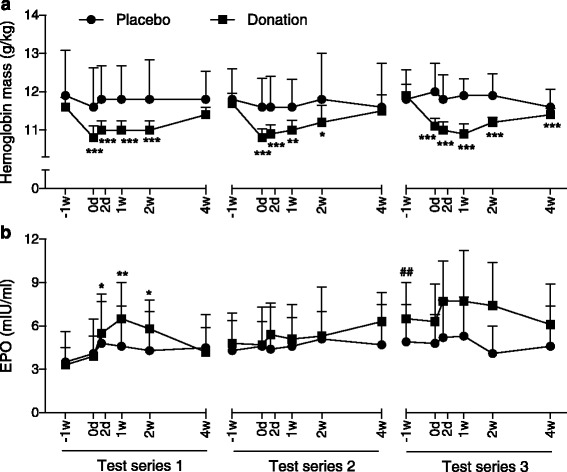
Hemoglobin mass and serum erythropoietin. Evolution of the hemoglobin mass (**a**) and serum erythropoietin (EPO, **b**) levels before and after each of the three test series in the placebo and donation groups. Data are expressed as means ± SEM. *n* = 3 in placebo and *n* = 7 in donation for test series 1, 2, and 3. ^##^ *p* < 0.01 vs. -1w of test series 1, same group. *p < 0.05, **p < 0.01, ***p < 0.001 vs. -1w of the same test series, same group

A single blood donation reduced hemoglobin mass by 7% the day of the donation and by 5% until 2 weeks after (Fig. 4a). Pre-donation values were recovered after 4 weeks. The second and the third donations lead to similar reductions in hemoglobin mass except that 4 weeks after the third donation hemoglobin mass was still 4% lower than pre-donation values. No change in hemoglobin mass (Fig. 4a) or serum erythropoietin concentration (Fig. 4b) was observed in the placebo group. A general increase in serum erythropoietin over time was found in the donation group (Table 2). Values before beginning the third test series were higher than pre-values in the first test series (Fig. 4b). Within the first test series, erythropoietin concentration increased by 50% 2d, by 100% 1w, and by 75% 2w after donation. This within test series augmentation was not observed in test series 2 and 3.

### Whole Blood Donation Affects Key Hematological Parameters for Oxygen Transport: Additive Effect of Repetitions

A global group effect was found for hematocrit, hemoglobin concentration, ferritin, and RBC (Table 2). A clear difference was observed between the placebo and the donation group in the evolution of those four parameters, all of which being important for oxygen transport. The regulation of hematocrit, hemoglobin concentration, ferritin, and RBC after each blood donation was similar to the regulation of hemoglobin mass, i.e., no difference in the placebo group and a decrease within each test series in the donation group. The maximal decrease after a blood donation observed in each parameter was 11% for hematocrit, 10% for hemoglobin concentration, 50% for ferritin, and 12% for RBC (Table 4). Contrary to hemoglobin mass, which recovered basal values before beginning the second and the third test series, values before test series 2 and 3 for hematocrit, hemoglobin concentration, ferritin, and RBC were all lower compared to values before test series 1 in the donation group (Table 4). Those results indicate that repeated blood donations decreased the levels of hematocrit, hemoglobin concentration, ferritin, and RBC more than one single donation. Serum iron levels (Table 4) and transferrin saturation (additional file 1) followed a similar pattern as the aforementioned parameters but the variation between subjects was higher, leading to somewhat less numerous significant decreases. Values for MCH and MCHC decreased and values for RDW increased over time in each group (Table 2). The amplitude of the decreases was small (max 4% for MCH and MCHC and 5% for RDW) but very consistent within each subject (Table 4). A general time effect was observed for MCV, MPV, and platelets but only in the donation group (Table 2 and additional file 1). WBC levels were not modified by any condition (additional file 1).

**Table 4:** Effect of repeated blood donations on hematological parameters

		Test series 1					Test series 2					Test series 3				
		-1w	2d	1w	2w	4w	-1w	2d	1w	2w	4w	-1w	2d	1w	2w	4w
Hematocrit	P	46.6 ± 1.2	46.4 ± 0.6	46.1 ± 1.0	46.4 ± 1.3	46.1 ± 1.1	44.5 ± 1.5	45.3 ± 1.5	44.7 ± 1.4	46.2 ± 1.5	46.3 ± 1.5	43.5 ± 1.6	45.9 ± 1.2	46.0 ± 1.2	45.9 ± 0.9	45.3 ± 1.5
(%)	D	45.8 ± 0.6	40.9 ± 0.9***	41.9 ± 0.6***	42.4 ± 0.5***	43.9 ± 0.5**	44.5 ± 0.5###	42.2 ± 0.6***	41.0 ± 0.5***	43.0 ± 0.4**	43.2 ± 0.5**	43.8 ± 0.5###	41.7 ± 0.7***	41.9 ± 0.6***	42.1 ± 0.7***	43.4 ± 0.5
Hemoglobin	P	15.9 ± 0.6	15.7 ± 0.3	15.5 ± 0.5	15.4 ± 0.6	15.3 ± 0.5	15.0 ± 0.7	15.0 ± 0.7	14.9 ± 0.7	15.4 ± 0.7	15.3 ± 0.7	14.7 ± 0.8	15.2 ± 0.6	15.2 ± 0.6	15.2 ± 0.5	15.3 ± 0.8
(g/dl)	D	15.6 ± 0.2	13.7 ± 0.3***	14.2 ± 0.2***	14.2 ± 0.2***	14.5 ± 0.2***	14.9 ± 0.2###	14.0 ± 0.3***	13.7 ± 0.2***	14.2 ± 0.2***	14.4 ± 0.2**	14.6 ± 0.2###	13.7 ± 0.3***	14.1 ± 0.2**	14.1 ± 0.3**	14.5 ± 0.2
Iron	P	112 ± 20	101 ± 18	83 ± 17	98 ± 30	99 ± 20	85 ± 8	99 ± 35	78 ± 23	82 ± 11	110 ± 27	84 ± 19	136 ± 31	85 ± 13	133 ± 15	127 ± 36
(μg/dl)	D	106 ± 9	90 ± 8	74 ± 8**	66 ± 7***	96 ± 6	115 ± 20	101 ± 13	66 ± 7**	81 ± 10*	69 ± 6**	97 ± 9#	109 ± 9	76 ± 10	82 ± 11	99 ± 12
Ferritin	P	72 ± 12	64 ± 14	56 ± 11	53 ± 11	58 ± 11	64 ± 16	50 ± 11	68 ± 18	64 ± 16	59 ± 11	77 ± 22	71 ± 17	82 ± 15	76 ± 15	69 ± 13
(μg/l)	D	55 ± 8	45 ± 7*	43 ± 8*	37 ± 8***	27 ± 5***	40 ± 6##	42 ± 7	27 ± 4**	28 ± 3*	23 ± 4**	41 ± 5###	37 ± 5	30 ± 5***	29 ± 4***	33 ± 5**
MCH	P	29.9 ± 0.6	29.7 ± 0.6	29.4 ± 0.6***	29.4 ± 0.7**	29.4 ± 0.6**	29.9 ± 0.5###	29.4 ± 0.5	29.8 ± 0.5	29.9 ± 0.5	29.5 ± 0.6	29.8 ± 0.7##	29.6 ± 0.6	29.5 ± 0.5	29.6 ± 0.5	30.0 ± 0.6
(pg)	D	30.0 ± 0.3	29.6 ± 0.4**	29.7 ± 0.4**	29.4 ± 0.3***	29.5 ± 0.4***	29.4 ± 0.4###	29.3 ± 0.4	29.4 ± 0.4	29.3 ± 0.4	29.1 ± 0.4	29.0 ± 0.4##	28.6 ± 0.4**	29.3 ± 0.4*	29.0 ± 0.5	29.1 ± 0.4
MCHC	P	34.1 ± 0.4	33.7 ± 0.4*	33.6 ± 0.5***	33.2 ± 0.5**	33.1 ± 0.4**	33.6 ± 0.5##	33.1 ± 0.6*	33.3 ± 0.6	33.3 ± 0.5	33.0 ± 0.6*	33.8 ± 0.8###	33.1 ± 0.7	33.0 ± 0.6	33.2 ± 0.6	33.6 ± 0.7
(g/dl)	D	34.0 ± 0.2	33.6 ± 0.2**	33.8 ± 0.2*	33.4 ± 0.2***	33.1 ± 0.3***	33.6 ± 0.3###	33.2 ± 0.3	33.4 ± 0.2	33.1 ± 0.2*	33.3 ± 0.3	33.4 ± 0.3##	32.7 ± 0.3***	33.6 ± 0.2	33.3 ± 0.3	33.4 ± 0.3
RDW	P	12.9 ± 0.2	12.9 ± 0.2	13.0 ± 0.2	13.2 ± 0.2	13.2 ± 0.2*	13.1 ± 0.3#	13.2 ± 0.4	13.1 ± 0.4	13.2 ± 0.4	13.1 ± 0.3	13.2 ± 0.5##	13.4 ± 0.5	13.4 ± 0.5	13.4 ± 0.5	13.4 ± 0.6
(%)	D	12.8 ± 0.1	12.7 ± 0.1	12.8 ± 0.1	12.9 ± 0.1	13.0 ± 0.1*	12.6 ± 0.1	12.7 ± 0.1**	13.0 ± 0.1***	12.9 ± 0.1	12.8 ± 0.1	13.2 ± 0.1###	13.4 ± 0.2	13.3 ± 0.1	13.4 ± 0.1	13.1 ± 0.1
RBC	P	5.3 ± 0.2	5.3 ± 0.1	5.3 ± 0.2	5.3 ± 0.2	5.2 ± 0.2	5.0 ± 0.2	5.1 ± 0.2	5.0 ± 0.2	5.1 ± 0.2	5.2 ± 0.1	5.0 ± 0.2	5.2 ± 0.1	5.2 ± 0.1	5.1 ± 0.1	5.1 ± 0.2
(10^12^/l)	D	5.2 ± 0.1	4.6 ± 0.1***	4.8 ± 0.1***	4.8 ± 0.1***	4.9 ± 0.1**	5.1 ± 0.1###	4.8 ± 0.1***	4.7 ± 0.1***	4.9 ± 0.1**	4.9 ± 0.1*	5.1 ± 0.1###	4.8 ± 0.1***	4.8 ± 0.1***	4.8 ± 0.1***	5.0 ± 0.1

Values are means ± SEM (*n* = 7 in *P* and *n* = 13 in *D* for test series 1; *n* = 5 in *P* and *n* = 13 in *D* for test series 2 and 3. *MCH* mean corpuscular hemoglobin, *MCHC* mean corpuscular hemoglobin concentration, *RDW* red blood cell distribution width, *RBC* red blood cell count, *P* placebo, *D* donation. ^#^ *p* < 0.05, ^##^ *p* < 0.01, ^###^ *p* < 0.001 vs. -1w of test series 1, same group. *p < 0.05, **p < 0.01, ***p < 0.001 vs. -1w of the same test series, same group

## Discussion

The key findings of the present study are that (1) maximal power output, VO_2_peak, and hemoglobin mass decreased up to 4 weeks after a single whole blood donation in moderately trained people; (2) training adaptations seemed somewhat lowered by repeated whole blood donations as endurance capacity did not increase following multiple maximal exercise tests in the donation while it did in the placebo group; (3) key hematological parameters for oxygen transport were lowered by a single whole donation and cumulatively further affected by the repetition of the donations; and (4) submaximal lactate levels, reflecting submaximal endurance capacity, were not modified by whole blood donation.

For the first time, a comprehensive study was performed including repeated blood donations and assessment of endurance capacity, hematological parameters, and hemoglobin mass by the optimized CO rebreathing method, with a follow-up of 4 weeks after each donation. In addition, a placebo group was included, which strongly increased the power of the study and thereby, the interpretation of the results. Except one study [28], all others failed to include such group and the comparisons were made before and after blood donation. When looking at short-term adaptations of a few hours or days, the lack of a placebo group is less critical and a pre-post analysis can be considered as sufficient. When changes are expected on the long term, the inclusion of a placebo group is mandatory, as the time between two measurements becomes a variable by itself. In the present case, the placebo group allowed the distinction between the effects caused by blood donation and the effects caused by the repetition of the exercise tests, which was expected to induce a global training effect. A typical example is the improvement of the maximal power output during test series 2 and 3 in the placebo group only. Without a placebo group, we could have concluded that a single donation alters maximal power output while repeated donations did not. The results of the placebo group indicate that the normal response to 15 maximal incremental tests is a global improvement in maximal power output. The conclusion is therefore that repeated blood donations reduce adaptations to training, at least at the level of maximal power output. On the other hand, the decrease in VO_2_peak in the donation group during the first test series is not totally attributable to blood donation as a decrease was observed after 1 and 4 weeks in the placebo group as well. It is therefore likely that the decrease in VO_2_peak we observed during the first test series was not totally attributable to donation itself but also to fatigue induced by repeated exercise tests. Interestingly, not only endurance parameters were affected by the repetition of the exercise tests, MCH, MCHC, and RDW were also training-sensitive, independently of the group. All together those results highlight the importance of including a placebo group when studies are performed on the long term.

We found a reduced maximal power output and VO_2_peak of a few percent up to 4 weeks after the first blood donation. The largest reduction in maximal power output was 4% and in VO_2_peak 11%, which corresponds to values previously reported [15, 20, 22, 24]. Only one study extended the recovery period until 4 weeks [15]. The main results of that study were that running performance on a 3-km time test series and VO_2_peak were reduced up to 1 week and hemoglobin concentration up to 2 weeks. In another study, VO_2_peak was shown to be reduced up to 2 weeks after donation while time to fatigue was not affected [24]. Here, we used a similar exercise protocol, i.e., maximal incremental test on a cycle ergometer, to determine endurance capacity and VO_2_peak. It is therefore surprising that endurance capacity was differently affected after blood donation. It is possible that the training status influences the effect of donation on endurance capacity as our subjects had an averaged VO_2_peak of 57 mlO_2_/kg/min and in Judd et al. a VO_2_peak of 47 mlO_2_/kg/min. One could postulate that somewhat better endurance-trained athletes will have a different reaction to blood donation than less well trained and could develop iron deficiency more rapidly as they usually have a low iron status. To confirm this hypothesis, subjects with different endurance training levels should be tested in the same study design but elite athletes are not willing to undergo blood donation, knowing the negative impact on their performance, even if temporary. The reduction in performance and VO_2_peak after blood donation is probably due to the limitation of blood oxygen transport capacity. Hematocrit, hemoglobin concentration, hemoglobin mass, ferritin, iron, and RBC were all reduced after blood donation and their values were still lower than basal values after 4 weeks, except for iron and hemoglobin mass. We hereby confirm previous reports showing that recovery of hematological parameters may take a few weeks or even more [15–17]. Here, hematocrit, hemoglobin concentration, ferritin, and RBC were still lower 3 months after the first blood donation, at the time of beginning the second test series, indicating that the recovery of those parameters was incomplete before the next blood donation. It is therefore not surprising to observe an additive effect of repeating blood donation on those parameters.

To the best of our knowledge, no study has prospectively analyzed the impact of repeated blood donations on endurance capacity. We found that maximal power output was mainly affected after the first donation with a minor impact after the second and the third one. Nevertheless, the expected improvement in maximal power output after repeated exercise tests, as observed in the placebo group, was not present in the donation group. It seems therefore that training adaptations are impaired due to repeated donations, probably due to a decrease in key hematological parameters. Hematocrit, hemoglobin concentration, ferritin, and RBC all decreased globally over time, not only within one test series. We hereby extend the results of a previous study looking at the effect of repeated blood donations on serum ferritin concentrations [17]. Ferritin levels were directly inversely proportional to the number of donations per year, with the lowest concentrations measured in people who gave blood thrice a year [17]. Of note, our subjects did not receive any iron supplementation as they had a normal iron status at baseline and were not considered at risk for anemia. Our results show that even in a population a priori without risk for iron deficiency, it would be interesting to consider an orientation of the diet towards an iron-rich diet and/or iron supplementation to try to limit the negative effect of repeated blood donations on iron status. At the same time, this could limit the reduction in endurance performance. Further investigation should therefore focus on possible countermeasures to limit the side effects of repeated blood donation on iron status and endurance performance. Of note, our subjects were not sensu stricto iron deficient as their ferritin levels were above 15 μg/l (World Health Organization) but it should be acknowledged that endurance performance can be altered before iron deficiency really takes place.

Measurement of hemoglobin and ferritin concentration as well as hematocrit to assess hematological recovery after blood donation may not reflect the true amount of blood as both are affected by changes in plasma volume. Instead, total hemoglobin mass has been proposed to be the most sensitive variable to assess hematological recovery [16]. After one blood donation, total hemoglobin mass has been shown to be reduced by about 8–9% and to recover pre-donation values after 35 days in average [16]. Of note, this recovery period was highly variable, ranging from 20 to 59 days. Here, we found similar amplitude in the decrease in total hemoglobin mass, which was recovered after 4 weeks in test series 1 and 2 but took more than 4 weeks in test series 3, indicating that the recovery period was longer when the donations were repeated. Concomitantly, serum erythropoietin levels were higher during the third donation, slightly increasing over time from the first measurement to the end of the study in the donation group. Erythropoietin is a critical hormone in the formation of red blood cells in the bone marrow, thereby increasing total hemoglobin mass [29]. Taken together, the evolution of hemoglobin mass and erythropoietin suggests that the regenerating capacity of total hemoglobin mass was slower with the repetition of blood donations. Interestingly, the decrease in hemoglobin mass was preceded by a decrease in hematocrit, hemoglobin concentration, RBC, and ferritin. As the quantification of the latter parameters is performed in routine when donating blood, a continuous decrease in their level could be interpreted as a premature sign of a later decrease in hemoglobin mass, which is more complex to assess. As hematological parameters are key factors in the adaptation to endurance exercise [14, 30], it is not surprising to see no improvement of maximal power output in the donation group while this was the case in the placebo group with the repetition of the maximal efforts. Notably, blood lactate concentrations at 190 W, which reflect submaximal training adaptations, decreased similarly over time in both groups. We hereby confirm a previous report showing that submaximal endurance capacity is not altered by blood donation [21].

The interpretation of the present results is limited to the population we studied, i.e., moderately trained people. As mentioned above, one can speculate that athletes with higher hemoglobin mass and VO_2_peak [31] would suffer greater losses in performance from blood donation. In addition, at the beginning of the study, half of the subjects were regular donors (three times a year) while the other half were not (one time or less a year). As already shown retrospectively in male Saudi blood donors, the higher the frequency of blood donations, the lower the ferritin concentrations [32], and probably the higher risk of developing iron deficiency. Here as well, regular donors had lower ferritin concentrations at the beginning of the study than non-regular donors (data not shown). However, no difference was observed between those two groups in the drop of Pmax or VO_2_peak after the first donation, indicating that the antecedent of the donors had a limited impact on our main outcome, endurance capacity. Another limitation of the present study is that we did not determine the anaerobic threshold and as a consequence no distinction could be made between the aerobic and anaerobic muscular work. Whether blood donation induces a shift in the anaerobic threshold due to reduced capacity for carrying oxygen remains to be tested.

Practically, the changes we observed in maximal power output and VO_2_peak were rather limited for a moderately trained population. Endurance capacity was reduced by a few percent for 4 weeks, which should not prevent moderately trained athletes to give blood as other uncontrolled factors such as the form of the day, a poor hydration status, motivation, work-induced fatigue, or familial emergency would have an impact of the same magnitude on their performance. We can recommend to those athletes not to plan an important race the month following a blood donation or not to donate blood the month before an important race. For elite athletes, blood donation is not to be recommended for the direct effect on endurance performance but also for the impairment repeated donations have on training adaptations. Reducing performance by a few percent can have dramatic consequences on the ranking. But this concerns only a small proportion of the total number of people practicing physical activity and participating in sport events.

## Conclusions

The present study is the first to look at the effect of repeated whole blood donation on endurance capacity and key hematological parameters for oxygen transport, among which is hemoglobin mass. We found that maximal, but not submaximal, endurance capacity was altered after blood donation in moderately trained people and that the expected training effects were lower when repeating blood donations. Those alterations are probably partially due to reductions in the iron status, red blood cells, and hemoglobin mass. Measures to counteract the alterations in hematological parameters should be developed to minimize alterations in endurance capacity and thereby to attract more athletes to become donors. Finally, athletes constitute a very healthy potential donor population and should consider becoming plasma donors as there also is an increased need for plasma worldwide and plasma donation does not affect their hemoglobin levels at all.

## Additional file

Additional file 1: Effect of repeated blood donations on hematological parameters. (DOCX 72 kb)
