# Supplementary material for: Effect of Repeated Whole Blood Donations on Aerobic Capacity and Hemoglobin Mass in Moderately Trained Male Subjects: A Randomized Controlled Trial
Source: Sports Med Open. 2016 Nov 22;2:43. doi: 10.1186/s40798-016-0067-7 (PMC5118378; doi:10.1186/s40798-016-0067-7)
Supplement: Additional file 1: — Effect of repeated blood donations on hematological parameters. (DOCX 72 kb) [file 40798_2016_67_MOESM1_ESM.docx]

Additional file 1. *Effect of repeated blood donations on hematological parameters*

|  |  | **Test series 1** | | | | | **Test series 2** | | | | | **Test series 3** | | | | |
| --- | --- | --- | --- | --- | --- | --- | --- | --- | --- | --- | --- | --- | --- | --- | --- | --- |
|  |  | **-1w** | **2d** | **1w** | **2w** | **4w** | **-1w** | **2d** | **1w** | **2w** | **4w** | **-1w** | **2d** | **1w** | **2w** | **4w** |
| **Transferrin** | P | 2.74±0.16 | 2.71±0.20 | 2.73±0.14 | 2.66±0.20 | 2.70±0.14 | 2.65±0.16 | 2.76±0.27 | 2.67±0.20 | 2.80±0.16 | 2.83±0.23 | 2.61±0.19 | 2.70±0.20 | 2.68±0.19 | 2.65±0.19 | 2.62±0.20 |
| (g/l) | D | 2.94±0.12 | 2.83±0.12* | 2.96±0.12 | 2.93±0.11 | 3.04±0.14* | 3.02±0.14 | 2.88±0.08 | 2.99±0.13 | 3.07±0.13 | 3.14±0.16 | 2.94±0.11 | 2.84±0.14 | 2.91±0.15 | 2.94±0.10 | 3.12±0.15 |
| **Transf sat** | P | 31±7 | 29±7 | 23±6 | 30±12 | 28±7 | 24±4 | 27±13 | 24±10 | 21±4 | 31±11 | 24±7 | 39±12 | 24±5 | 36±4 | 37±14 |
| (%) | D | 26±2 | 23±2 | 18±2* | 16±2** | 23±2 | 27±4 | 25±3 | 16±2** | 19±2* | 16±2** | 24±3# | 28±2 | 19±3 | 21±3 | 23±3 |
| **MPV** | P | 12.1±0.3 | 11.5±0.3* | 12.0±0.3 | 11.9±0.4 | 11.6±0.2* | 11.9±0.2 | 11.7±0.5 | 12.2±0.4 | 12.1±0.4 | 12.1±0.4 | 11.9±0.4 | 12.1±0.4 | 12.0±0.4 | 12.2±0.4 | 12.0±0.5 |
| (fl) | D | 11.3±0.2 | 11.0±0.3*** | 11.0±0.3** | 11.0±0.3** | 11.2±0.2 | 11.4±0.2 | 11.4±0.2 | 11.3±0.3 | 11.3±0.3 | 11.3±0.3 | 11.3±0.2 | 11.4±0.3 | 11.3±0.3 | 11.2±0.3 | 11.5±0.3 |
| **MCV** | P | 87.6±1.1 | 87.9±1.0 | 87.5±1.1 | 88.3±1.4 | 88.8±1.3** | 88.8±0.7 | 88.7±1.4 | 89.4±1.0 | 90.0±0.8 | 89.3±0.7 | 88.1±0.4 | 89.2±0.5 | 89.2±0.5 | 89.4±0.5* | 89.5±0.9* |
| (fl) | D | 88.2±0.7 | 88.1±0.9 | 87.8±0.8* | 88.1±0.7 | 88.9±0.8*** | 87.7±0.8 | 88.3±0.7 | 87.7±0.8 | 88.6±0.8 | 87.4±0.8 | 86.9±0.8 | 87.3±0.9* | 87.2±0.8** | 86.9±0.8 | 87.0±0.9 |
| **WBC** | P | 6.05±0.58 | 6.05±0.59 | 6.33±0.82 | 5.95±0.56 | 6.01±0.74 | 5.19±0.44 | 6.10±0.75 | 5.57±0.64 | 5.56±0.70 | 5.44±0.62 | 6.21±0.99 | 5.69±0.88 | 5.57±0.72 | 6.52±1.03 | 5.79±0.96 |
| (10^9^/l) | D | 5.62±0.35 | 5.55±0.34 | 6.28±0.39 | 6.02±0.42 | 5.45±0.40 | 5.88±0.40 | 5.27±0.27 | 5.41±0.41 | 5.64±0.54 | 5.58±0.37 | 5.59±0.22 | 5.41±0.30 | 5.32±0.38 | 5.51±0.43 | 5.94±0.46 |
| **Platelets** | P | 238±13 | 240±17 | 252±27 | 238±26 | 250±15 | 233±12 | 208±11 | 244±15 | 227±14 | 235±10 | 196±12 | 229±17** | 222±16* | 209±14 | 210±13 |
| (10^9^/l) | D | 232±11 | 227±9** | 252±12** | 253±13 | 235±12 | 230±12 | 233±10 | 238±12 | 238±10 | 232±7 | 219±12 | 214±10 | 228±10 | 220±10 | 228±12 |

Values are means ± SEM (n=7 in P and n=13 in D for test series 1; n=5 in P and n=13 in D for test series 2 and 3). Transf sat, transferrin saturation; MPV, mean platelet volume; MCV, mean corpuscular volume; WBC, white blood cell count; P, placebo; D, donation. ^#^p<0.05, ^##^p<0.01, ^###^p<0.001 vs -1w of test series 1, same group. *p<0.05, **p<0.01, ***p<0.001 vs -1w of the same test series, same group.
